# Glucagon secretion and signaling in the development of diabetes

**DOI:** 10.3389/fphys.2012.00349

**Published:** 2012-09-04

**Authors:** Herbert Y. Gaisano, Patrick E. MacDonald, Mladen Vranic

**Affiliations:** ^1^Departments of Medicine and Physiology, University of TorontoToronto, ON, Canada; ^2^Department of Pharmacology, University of AlbertaEdmonton, AB, Canada

**Keywords:** islet α-cell, glucagon secretion, diabetes, hypoglycemia, somatostatin

## Abstract

Normal release of glucagon from pancreatic islet α-cells promotes glucose mobilization, which counteracts the hypoglycemic actions of insulin, thereby ensuring glucose homeostasis. In treatment of diabetes aimed at rigorously reducing hyperglycemia to avoid chronic complications, the resulting hypoglycemia triggering glucagon release from α-cells is frequently impaired, with ensuing hypoglycemic complications. This review integrates the physiology of glucagon secretion regulating glucose homeostasis *in vivo* to single α-cell signaling, and how both become perturbed in diabetes. α-cells within the social milieu of the islet micro-organ are regulated not only by intrinsic signaling events but also by paracrine regulation, particularly by adjacent insulin-secreting β-cells and somatostatin-secreting δ-cells. We discuss the intrinsic α-cell signaling events, including glucose sensing and ion channel regulation leading to glucagon secretion. We then discuss the complex crosstalk between the islet cells and the breakdown of this crosstalk in diabetes contributing to the dysregulated glucagon secretion. Whereas, there are many secretory products released by β- and δ-cells that become deficient or excess in diabetes, we discuss the major ones, including the better known insulin and lesser known somatostatin, which act as putative paracrine on/off switches that very finely regulate α-cell secretory responses in health and diabetes. Of note in several type 1 diabetes (T1D) rodent models, blockade of excess somatostatin actions on α-cell could normalize glucagon secretion sufficient to attain normoglycemia in response to hypoglycemic assaults. There has been slow progress in fully elucidating the pathophysiology of the α-cell in diabetes because of the small number of α-cells within an islet and the islet mass becomes severely reduced and inflamed in diabetes. These limitations are just now being surmounted by new approaches.

## Overview

Glucagon is a counter-regulatory hormone that counteracts insulin by promoting glucose mobilization in liver through glycogenolysis and gluconeogenesis (Dunning and Gerich, 2007; Quesada et al., 2008). Normally, low glucose levels trigger glucagon release from α-cells to abrogate the deleterious effects of insulin-induced acute hypoglycemia (Cryer, 2002; Dunning and Gerich, 2007; Quesada et al., 2008). As described below, even the very question of whether low blood sugar directly triggers glucagon release remains unresolved. In Type 1 (T1D) and Type 2 diabetes (T2D), glucagon secretion from α-cells become dysregulated (Gerich et al., 1973; Bolli et al., 1983; Butler and Rizza, 1991; Cryer, 2002; Dunning and Gerich, 2007; Quesada et al., 2008). In early diabetes, α-cells hypersecrete during meals (Butler and Rizza, 1991; Dunning and Gerich, 2007; Quesada et al., 2008) causing excessive release of glucose, resulting in hyperglycemia and eventually consequent chronic complications (blindness, kidney, heart failure, and neuropathy). As diabetes progresses, becoming more severe, more β-cells die and α-cells suffer from “hypoglycemic blindness” (perhaps partly due to increased sensitivity to exogenous insulin), becoming sluggish in response to low glucose, exacerbating life-threatening acute hypoglycemic episodes, responsible for ~5% of mortality due to diabetes (Gerich, 1988; Cryer, 2002). About 20 million people world-wide are at risk of developing hypoglycemia, of which 9 million reside in North America. It is well known that on average, insulin-treated diabetic patients have 2–3 hypoglycemic events per week. Hypoglycemia is one of the key acute complications of insulin therapy in diabetes treatment.

In addition to increased α-cell sensitivity to insulin, chronic hyperglycemia plays an important role in dysregulation of α-cells (Shi et al., 1996) but no effective treatment has been developed to increase (or regulate) glucagon secretion to combat hypoglycemic attacks. The primary reason for the lack of evidence is the very low-yield isolation (~20% of islet cells) and unreliable identification of islet α-cells, which severely impairs accuracy and efficiency in studying α-cell biology. In contrast, tremendous efforts have been directed at studying islet β-cell biology (Henquin et al., 2003; Rorsman and Renstrom, 2003), leading to development of effective drug compounds to stimulate insulin secretion (Moller, 2001) and improved islet transplantation strategies (Shapiro et al., 2000). The biggest obstacle in islet transplantation is limited supply of donor islets, thus great efforts have been directed at strategies of “pure” β-cell replacement derived from stem cells (Ramiya et al., 2000) or surrogates (Newgard, 1994)—the panacea of the “cure” for diabetes! Of note, not only β-cell but also α-cell mass in transplanted islets are reduced, with surviving α-cells suffering from defective response to glucose and incretin hormone glucagon-like peptide-1 (GLP-1) (Newgard, 1994; Gupta et al., 1997; Paty et al., 2002; Zhou et al., 2008; Rickels et al., 2009).

In spite of severe technical restrictions, the importance of α-cell in islet biology, pathobiology and implication on cell replacement therapy of T1D have been the impetus of the vigorous thrust of key labs to examine the intimate cross-talk between islet cells (Ishihara et al., 2003), α-cell glucose-sensing (Heimberg et al., 1996), α-cell ion channels (Kanno et al., 2002), and glucagon exocytosis (Barg, 2003) not only in rodent models, but also in human α-cells (MacDonald et al., 2007; Rorsman et al., 2008). Since, islet α-cell mass remains relatively intact even in advanced stages of diabetes (Stefan et al., 1982; Rahier et al., 1983), native α-cells lend itself to pharmacological intervention (Dunning et al., 2005; Dunning and Gerich, 2007). However, the precise molecular regulators by which glucose couples to secretory components of α-cells remain hotly debated (Gromada et al., 2007; Quesada et al., 2008). How these coupling mechanisms become dysregulated in diabetes albeit recently postulated (switch on/off hypothesis), the precise underlying cellular basis is unknown.

## The role of glucagon in diabetes

Roger Unger postulated that diabetes is caused by insulin deficiency (amount or effect) and glucagon presence, or excess (Unger et al., 1970). The main problem with Unger's hypothesis was that diabetes can be induced in animals either by selective chemical destruction of insulin producing β-cells, or by total pancreatectomy, which removes both β-cells and glucagon-producing α-cells, and diabetes occurs in both protocols irrespective of whether the α-cells have been removed by pancreatectomy. If glucagon is essential for development of diabetes, one would have expected that total pancreatectomy would not induce diabetes. But that was not the case. At that time, the laboratories that were best known for measuring glucagon in plasma were those of Roger Unger in Dallas Texas, Pierre Lefebvre in Liege, and Roger Assan in Paris. All three laboratories reported that after pancreatectomy in dogs, plasma glucagon could not be detected. A few years earlier, two Nobel Prize winners, DeDuve and Sutherland, showed that extracts from the mucosa of the dog's stomach have a hyperglycemic effect (Sutherland and De Duve, 1948). This could have been glucagon, but quantitative glucagon assay did not yet exist. To our surprise and in contrast to other laboratories, we could still detect plasma glucagon in insulin-infused depancreatized dogs. If insulin treatment was discontinued, glucagon skyrocketed within a week after pancreatectomy (Vranic et al., 1974). These results created a great deal of excitement because it was contrary to the dogma that depancreatized dogs did not have any glucagon in the plasma. This led to the stunning discovery, that a pancreatic hormone can be produced in large amounts outside its original endocrine gland. Over the next 10 years, using tracer methods to measure glucose fluxes, biochemical, histological, immunological methods, electron microscopy, and purifying gastric glucagon to homogeneity, it was determined beyond all doubt that the parietal mucosa of the dog stomach can synthesize and secrete true glucagon (Morita et al., 1976; Doi et al., 1979).

When stomach glucagon was purified to homogeneity, its effect on isolated liver cells *in-vitro* was quantified. The effects of the extracts were identical to those of pancreatic glucagon. Now, it was not surprising by measuring glycogenolysis, gluconeogenesis, production of lactate and pyruvate, and concentration of cAMP, that following pancreatectomy in dogs, diabetes is as severe as with the selective destruction of the β-cells (Doi et al., 1979). Another stunning finding was that in the gastric mucosa of a depancreatized dog that was maintained on insulin by for 5 years, there was a large hyperplasia of α-cells, and a large amount of glucagon in the dog's stomach. By electron microscopy of the parietal mucosa of the stomach looked like a glucagon-producing endocrine gland (Ravazzola et al., 1977). It was demonstrated with labeled tryptophan, leucine, and s-methionine, the specific biosynthesis of glucagon in mucosa pieces of the stomach (Hatton et al., 1985). These findings challenged classical views of endocrinology and provided further proof that one hormone is not necessarily produced in only one endocrine gland. Furthermore, the amount of glucagon-like peptides that are secreted exclusively from the gastro-intestinal tract was quantified (Mojsov et al., 1987). High glucagon plasma levels in the depancreatized dogs were also confirmed by others (Matsuyama and Foa, 1974). Their regulation of extrapancreatic glucagon release was different than that from the pancreas (Luyckx and Lefebvre, 1983). True glucagon was localized exclusively in the stomach because pancreatectomy plus gastrectomy virtually removed glucagon from plasma (Muller et al., 1978). The most extensive factors that control gastric glucagon release were ascertained by using a unique model of isolated-perfused dog stomach (Lefebvre and Luyckx, 1977). Arginine elicited rapid gastric glucagon release. This glucagon release was almost completely abolished by somatostatin. It was not affected by hypoglycemia alone, but was reduced by 40% when hyperglycemia was concomitant with hyperinsulinemia. Thus, insulin is needed for hyperglycemia to inhibit gastric glucagon secretion. Perfused dog stomach provides a unique tool for investigating α-cell function in absence of endogenously released insulin. In addition, they also reported that immune-neutralization of insulin in the blood perfusing the stomach doubled the glucagon release, and thus further confirmed the role of insulin in controlling α-cell secretion (Lefebvre and Luyckx, 1978). These early observations in the dog stomach are relevant in the studies of pancreatic slices, of streptozotocin (STZ) and BioBreeding (BB) diabetic rats, which will be reported later in this review. In contrast to dogs, in totally depancreatized humans, there is only a negligible amount of plasma glucagon, and in contrast to depancreatized dogs, in depancreatized humans, diabetes is very mild (Barns et al., 1977; Muller et al., 1979; Boden et al., 1980; Holst et al., 1983). Thus, the discovery of extra-pancreatic glucagon led to a much better understanding of the role of glucagon in physiology and diabetes.

Glucagon-like peptides are detected in the brain (Tager et al., 1980; Tominaga et al., 1981; Hatton et al., 1982) and that stimulated interest in this field. The discovery of extra-pancreatic glucagon and quantification of release of glucagon-like peptides from the intestine, also stimulated research in the field of GLP-1 that is co-encoded in the glucagon gene as a potent stimulator of insulin release (Mojsov et al., 1987; Drucker, 2005).

Recently, studies in glucagon receptor-null mice (*Gcgr*^−/−^) indicate that glucagon mediates the metabolic consequences of insulin lack (Lee et al., 2011). In these mice, which exhibit no response to glucagon at any concentration, destruction of β-cells did not result in any of the diabetic abnormalities thought to be caused by insulin deficiency. Destruction of β-cells in wild-type controls resulted in the familiar catabolic consequences of insulin deficiency, with death due to ketoacidosis within 6 weeks, whereas in the *Gcgr*^−/−^ mice, none of the clinical or laboratory manifestations of insulin deficiency was detected. The insulin-deficient *Gcgr*^−/−^ mice did not become hyperglycemic or hyperketonemic, and their livers exhibited no increase either in phosphor-cAMP response element-binding protein (p-CREB); a mediator of glucagon action (Altarejos and Montminy, 2011) or in the gluconeogenic enzyme phosphoenolpyruvate carboxykinase, both of which are elevated in uncontrolled diabetes. Unquestionably, this exciting new finding indicates an important role of glucagon in diabetes. The interesting question is whether there are compensatory mechanisms that occur in knock-out rodents that replace the action of insulin, such as increased insulin-like growth factor (IGF)-1 or increased sensitivity of insulin receptors to IGF-1. It is also difficult with the methods presently used to ascertain that insulin has been completely removed. One could speculate that some knock-outs procedures may alter the physiology of insulin-glucagon interactions, and may reflect a metabolic system not seen in physiology or in diabetes.

## The cellular mechanism of α-cell glucagon secretion

**Figure 1:**
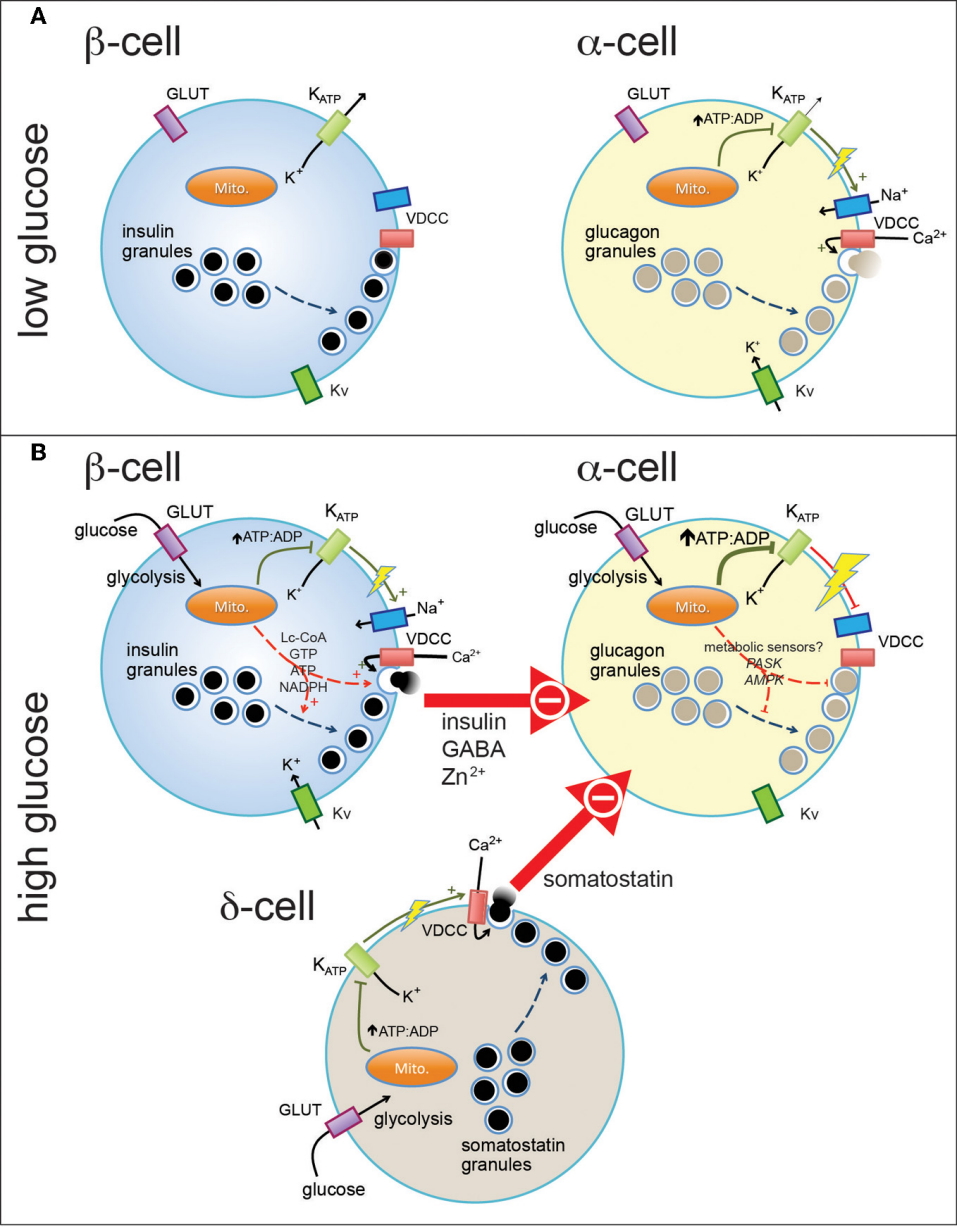
**Pancreatic endocrine cells are regulated by intrinsic and paracrine signals in response to glucose.** At basal glucose levels pancreatic β-cells (**A**, *left*) are electrically inactive, owing to their open ATP-sensitive K^+^ (K_ATP_) channels and resultant hyperpolarized membrane potential, and thus do not secrete insulin. In contrast, α-cells in the presence of low glucose (**A**, *right*) are electrically active, due to a relatively elevated ATP/ADP ratio (even at low glucose). This results in closure of most, but perhaps not all, K_ATP_ channels and a depolarized membrane potential that allows action potential firing mediated by a combination of voltage-dependent Na^+^, Ca^2+^ (VDCC), and K^+^ (Kv) channels. Entry of Ca^2+^ through VDCCs triggers the exocytosis of glucagon-containing secretory granules. When plasma glucose is increased **(B)**, glucose enters pancreatic islet cells through plasma membrane glucose transporters (GLUT) where it is metabolized through glycolysis and mitochondrial oxidative metabolism. This results in increase in the intracellular ATP/ADP ratio (in the case of α-cells, a further increase) and closure of K_ATP_ channels. In the β-cell (**B**, *top left*) this results in membrane depolarization and firing of action potentials that, in combination with additional mitochondrial signals, results in the exocytosis of insulin-containing granules. In α-cells, further closure of K_ATP_ channels may further depolarize the membrane and lead to the depolarization-dependent inactivation of Na^+^ channels and VDCCs. Glucagon secretion is also inhibited by paracrine factors secreted from β-cells and pancreatic δ-cells. These signals may interact with putative α-cell metabolic sensors (i.e., PASK and AMPK) to produce the physiological suppression of glucagon secretion.

The cellular machinery that controls glucagon secretion from α-cells is perhaps surprisingly similar to that which regulates insulin secretion from β-cells (Figure 1). Glucagon-containing secretory granules, like those containing insulin, exist in what can be defined functionally as “reserve” and “releasable” pools. Like the secretion of insulin from β-cells, the exocytotic release of glucagon is triggered by Ca^2+^ entry through voltage-dependent Ca^2+^ channels (VDCCs) (Barg, 2003; Altarejos and Montminy, 2011). An increase in glucose decreases intracellular Ca^2+^ in α-cells (both isolated ones and within intact islets) (Olofsson et al., 2004; Cabrera et al., 2006; MacDonald et al., 2007; Vieira et al., 2007), although, this has been recently questioned in α-cells identified through α-cell-specific expression of fluorescent proteins (Le Marchand and Piston, 2010). Regardless, in response to increases in Ca^2+^, glucagon granule exocytosis is mediated by the action of exocytotic SNARE proteins that, like in β-cells, consists of a multi-protein complex including SNAP-25 and syntaxin 1A (Andersson et al., 2011). The insulin and glucagon secreting islet cells also share a principal exoctotic Ca^2+^ sensor, synaptotagmin VII (Gustavsson et al., 2009), although, as in β-cells (Gauthier and Wollheim, 2008) the involvement of other synaptotagmin isoforms has not been ruled out. The compliment of ion channels expressed in α-cells mirrors those found in β-cells. ATP-dependent K^+^ (K_ATP_) channels, voltage-dependent K^+^ (Kv) channels, and VDCCs are all present in both α- and β-cells (Ronner et al., 1993; Bokvist et al., 1999; Suzuki et al., 1999; MacDonald et al., 2007; Ramracheya et al., 2010; Spigelman et al., 2010). While the K^+^ channel isoforms may be largely the same in the two cell types, the relative contribution of VDCC isoforms is somewhat different in α-cells, with a greater contribution of P/Q-type channels in human α-cells [the details of α-cell Ca^2+^ signaling have been recently extensively reviewed (Rorsman et al., 2012)]. Additionally, voltage-dependent Na^+^ channels are thought to play a more prominent role in glucagon, rather than insulin, secretion (Gromada et al., 1997; Barg et al., 2000; Gopel et al., 2000; Ramracheya et al., 2010) and glucagon release is sensitive to the Na^+^ channel inhibitor tetrodotoxin (Gopel et al., 2000; Gromada et al., 2004; MacDonald et al., 2007).

It is not surprising then that pancreatic α-cells are electrically excitable and, like β-cells, use their electrical activity to couple changes in glucose to the regulation of glucagon release (Rorsman and Hellman, 1988; Gromada et al., 1997; Yoshimoto et al., 1999; Barg et al., 2000). Looking at this excitatory and exocytotic machinery alone however, is becomes difficult to explain how glucose inhibits, rather than stimulates, α-cell glucagon secretion. Understanding how the glucagon secretory machinery is regulated by signals both intrinsic and extrinsic to the α-cell will be necessary to elucidate the exact mechanism of glucose-regulated glucagon secretion. Indeed, there are already hints that the excitatory machinery in α-cells is regulated in a manner opposite to that of β-cells: for example membrane depolarization is capable of turning off a number of the ion channels involved in α-cell electrical activity that are activated under similar conditions in β-cells (Ramracheya et al., 2010; Spigelman et al., 2010); and hormonal signals, notably GLP-1, activates Ca^2+^ currents in β-cells (Salapatek et al., 1999) but inhibits these in α-cells (De Marinis et al., 2010). Thus, elucidating not only the pieces of machinery that control glucagon secretion, but how these are regulated will provide novel insight into the physiological mechanism for glucose-regulated glucagon release.

### Can glucose regulate glucagon secretion directly?

This question has been a matter of debate for many years. Based solely on studies of dispersed or purified α-cells (Pipeleers et al., 1985; Ishihara et al., 2003; Olsen et al., 2005; Le Marchand and Piston, 2010), the answer would seem to be no, since under these conditions glucose stimulates glucagon secretion. One must be quite careful in the interpretation of such studies however, since properties of both dispersed α- and β-cells are quite different than those in intact islets. For example, the presence of functional gap junction connections is recently proposed as necessary for the efficient suppression of glucagon secretion (Ito et al., 2012). These of course would be lost upon dispersion and purification of α-cells. Within α-cells, glucose certainly has metabolic effects (Detimary et al., 1998). While ATP levels may be high under low glucose conditions, studies using the cell-targeted ATP-sensor luciferase demonstrate a further increase (by 15%) in α-cell ATP in response to glucose (Ishihara et al., 2003; Ravier and Rutter, 2005). Recent evidence has implicated α-cell resident metabolic sensing in the control of glucagon secretion, and in the pathophysiology of glucagon secretion in diabetes through AMP-activated protein kinase (AMPK) (Leclerc et al., 2010) and Per-arnt-sim (PAS) domain-containing protein kinase (PASK) (da Silva Xavier et al., 2011). Glucose-dependent inhibition of glucagon secretion was associated with an inhibition of AMPK activity, while forced activation of AMPK stimulated glucagon secretion. This study (Leclerc et al., 2010) suggests then that although the baseline ATP level may be high, a glucose-stimulated rise in ATP (and drop in ADP and AMP) can indeed be sensed within the α-cell. This is particularly interesting since the AMPK pathway generally acts as a “master regulator” of energy metabolism. While the idea that AMPK activation may be beneficial in diabetes may seem at odds with the glucagon-stimulating effects of AMPK activation, this must be considered in the context of the activity of upstream AMPK regulators which themselves may be regulated by glucose in α-cells. At this time there is little or no information about the up- or down-stream regulators of AMPK in α-cells, although, this is currently an area of growing interest in the context of insulin secretion [reviewed in ref. Fu et al. (2012)]. Thus, the α-cell may indeed be capable of responding to intrinsic metabolic signals, and integrating these inputs with extrinsic paracrine signals in the physiologic control of glucagon secretion.

### Are α-cell K_ATP_ channels involved in the regulation of glucagon secretion?

The activity of α-cell K_ATP_-channels is thought to contribute to the control of glucagon secretion (Ronner et al., 1993; Bokvist et al., 1999; Gromada et al., 2004; MacDonald et al., 2007). A role for K_ATP_ channels in the regulation of glucagon secretion is supported by the reduced glucagon secretion under low-glucose conditions seen in mice lacking functional K_ATP_-channels (Gromada et al., 2004; Munoz et al., 2005; MacDonald et al., 2007), where increasing glucose stimulated glucagon secretion similar to that observed in purified α-cells. Although, α-cell K_ATP_ channels are thought to be ~94% inhibited already at low glucose conditions (Barg et al., 2000; Gopel et al., 2000; Gromada et al., 2004; Olsen et al., 2005), perhaps explaining the ability of α-cells to generate action potentials under this condition, experiments titrating K_ATP_ channel activity revealed a bell-shaped curve where glucagon release was inhibited by either too much or too little K_ATP_ conductance (MacDonald et al., 2007; Rorsman et al., 2008). However, it should be recognized that all K_ATP_-channel measurements in α-cells suggest that the effect of glucose on overall channel activity may be smaller than that seen with pharmacologic agents (Gromada et al., 2004; Olsen et al., 2005). Nonetheless, given the low input resistance of α-cells, small changes in K_ATP_ channel activity may be functionally relevant.

Interestingly, work in mice expressing GFP under the control of the mouse insulin promoter (MIP-GFP mice) showed that α-cell K_ATP_ channels are more sensitive to ATP than are those in β-cells (Leung et al., 2005, 2006). In those reports, we showed that insulin reduces the sensitivity of α-cell K_ATP_ channels to ATP relatively more so than β-cell K_ATP_ channels, and this was by its actions on the insulin receptor-phosphatidylinositol 3-kinase signaling pathway. This may account for the fact that most (92%) of α-cell K_ATP_ channels are closed at 1mM ATP. Since, the ATP concentration inside α-cell is >1 mM at low glucose (Vieira et al., 2007; Huang et al., 2011b), this could account for the large proportion of K_ATP_ channels that are closed in α-cells at low glucose and perhaps indicates that changes in other cytosolic factors [i.e., phospholipids (Baukrowitz et al., 1998; Shyng and Nichols, 1998) and glucose metabolites (Duchen et al., 1993; Mertz et al., 1996; Schuit et al., 1997; Dufer et al., 2002)] can modulate K_ATP_ channel sensitivity to ATP. The factors and signaling mechanisms that control α-cell K_ATP_ channel sensitivity to ATP are not well understood, although, regulation by paracrine factors may represent one such mechanism that could bridge the divide in understanding the interplay between paracrine and intrinsic factors controlling glucagon secretion.

### Complex crosstalk between islet cells

Release of islet hormones is regulated not only by direct actions of glucose and other nutrients, but also indirectly and potently by paracrine factors secreted by adjacent islet cells. The current body of knowledge shows that islet cells profoundly modulate each other's secretory functions by very complex paracrine and even autocrine pathways (Gaisano and Leung, 2008). High glucose stimulates β- ad δ-cell secretion while inhibiting α-cell secretion, whereas low glucose stimulates α-cell secretion directly or indirectly, but inhibits other islet cells (Dunning and Gerich, 2007; Quesada et al., 2008). Since insulin reduces K_ATP_ channel sensitivity to ATP in α-cells more so than β-cells (Leung et al., 2006), the sequential glucose-insulin paracrine control on α-cells via modulation of α-cell K_ATP_ channel sensitivity to ATP blockade likely becomes distorted in T2D, which would contribute to the dysregulated glucagon secretion. Indeed, glucagon secretion has long been known to be inhibited by insulin (Le Marchand and Piston, 2010; Andersson et al., 2011), but just now shown to be mediated *via* the α-cell-specific expression of the insulin receptor using a knockout strategy (Kawamori et al., 2009). Other β-cell secretory products also inhibit glucagon secretion, including Zn^2+^ (Ishihara et al., 2003; Franklin et al., 2005) and γ-aminobutyric acid (GABA) co-released from dense-core insulin granules and synaptic vesicles (GABA only) (Franklin and Wollheim, 2004; Wendt et al., 2004), together asserting redundant paracrine inhibition of glucagon secretion (Gromada et al., 2001; Cejvan et al., 2003; Franklin and Wollheim, 2004; Wendt et al., 2004; Franklin et al., 2005; Ludvigsen et al., 2007; Plöckinger and Strowski, 2007; Hauge-Evans et al., 2009; Kawamori et al., 2009). L-glutamate released from α- (Cabrera et al., 2008) and β-cells (Hayashi et al., 2003) stimulates GABA secretion from β-cells under low glucose, and acts directly on α-cells to stimulate glucagon release, thus asserting counteracting paracrine inhibitory and autocrine stimulatory actions on α-cells. Glucagon secreted by α-cells exhibits paracrine stimulatory action on β-cells and autocrine stimulation of α-cell glucagon secretion (Ma et al., 2005).

### Breakdown in islet crosstalk in diabetes

A major acute complication of diabetes is a defective response of glucagon, catecholamines and glucocorticoids to insulin-induced hypoglycemia coined “glucose blindness.” This occurs more frequently with antecedent hypoglycemia and hypoglycemia unawareness, and it is in part due to poor counterregulatory responses (Bolli et al., 1983; Dagogo-Jack et al., 1993; The Diabetes Control and Complications Trial Research Group, 1993; Fanelli et al., 1994). It is particularly important that some diabetes patients have increased risk of hypoglycemia during insulin treatment therapy (White et al., 1983). The threat of hypoglycemia has increased since the treatment for diabetes has aimed for tight blood glucose control to decrease the risk of diabetic complications. In order to avoid hypoglycemia, many diabetic patients reduce their blood glucose control. Thus, hypoglycemia is a limiting factor for proper control of glycemia. Therefore, it is important to develop a treatment strategy that would decrease the risk of hypoglycemia. The defect of glucagon and epinephrine responses to hypoglycemia in diabetes is puzzling because both counterregulatory responses are normal or even excessive during some stresses, such as moderate and strenuous exercise, both in dogs and humans (Orci et al., 1976; Wasserman et al., 1985; Marliss and Vranic, 2002). We showed that although in each islet the number of glucagon cells is greatly increased, the total amount of glucagon in the pancreas remains unchanged because of the reduction in the number of islet cells. Clearly, alloxan or STZ destroys not only β-cells, but they also reduce the total number of islet cells. It is well known that the release of glucagon by the pancreas is inhibited by both insulin and somatostatin; and in diabetes, defects in the release of these islet paracrine hormones contribute to the perturbation of glucagon release from α-cells.

Thus, the physiological regulation of glucagon secretion is complex (Figure 1). Under conditions of hypoglycemia the α-cell is electrically active, in part due to a high basal ATP (and consequent inhibition of much of the K_ATP_ currents), which allows opening of Ca^2+^ channels and glucagon exocytosis. At high glucose, paracrine inputs from both β- and δ-cells are crucial physiological suppressors of glucagon release through actions on the α-cell electrical and secretory machinery. Although controversial, metabolic sensing pathways intrinsic to the α-cell likely contribute to the suppression of glucagon release either directly, by inhibiting the α-cell ion channels and exocytotic machinery, or indirectly by modulating the cellular response to paracrine signals. As such, glucagon release is the result of an integrated α-cell response to external and internal cues. A breakdown in these mechanisms in diabetes likely contributes to hyperglucagonemia and impaired counterregulatory responses.

## Defective switches of insulin and somatostatin

### Defective insulin switch

In T1D, there is a lack of decrement changes in intraislet insulin occurring which has been postulated to account for the defective glucagon counter-regulation to hypoglycemia. α-cell sensitivity during hypoglycemia improves when normoglycemia is achieved by chronic phloridzin treatment, but not by insulin treatment in diabetic rats (Shi et al., 1996). This is partly due to insulin inhibition of glucagon synthesis and release (Liu et al., 1992; Amatruda and Livingston, 2003). This thinking became refined in two *in vivo* reports elegantly demonstrating the insulin “switch on-off” actions on α-cell and “glucose blindness” in glucagon secretory response in T1D (and T2D) (Paty et al., 2002; Hope et al., 2004). These reports showed the ability of low glucose to stimulate α-cell secretion requires initial increase in insulin levels (switch on) followed by insulin deprivation (switch off) in presence of low glucose. The intraislet insulin “switch off” hypothesis has however been further refined to suggest that it may not be due to insulin *per se* (Hope et al., 2004; Zhou et al., 2004), but rather Zn^2+^ (bound and co-released with insulin) (Zhou et al., 2007); but this notion was also challenged (Ravier and Rutter, 2005). Thus, the precise cellular/molecular mechanisms of insulin and Zn^2+^ switch on-off actions remain unclear.

### Defective somatostatin switch

**Figure 2:**
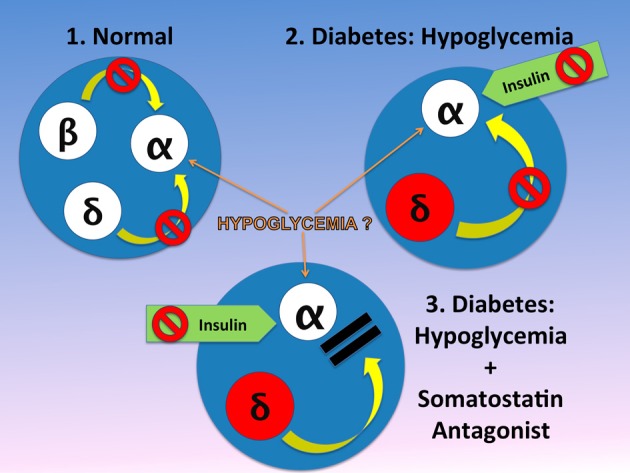
**In the normal physiology, the α-cell is under the tonic inhibitory influence of insulin and therefore somatostatin inhibition of α-cell may be of minor or no importance (Singh et al., 2007).** This is in contrast to diabetic islets in diabetes, where α-cell may be more sensitive to insulin and in addition, both circulating and pancreatic somatostatin, are increased. It is generally believed that hypoglycemia is a strong stimulator of glucagon release from the α-cell. However, in islets *in-vitro* the effect of hypoglycemia is not consistent. This difference may reflect the fact that between *in-vitro* and *in-vivo* systems, *in-vivo* the islets have abundant blood flow, which brings to the islet other factors such as amino acids (i.e., arginine) that can stimulate glucagon release. We hypothesize therefore, that hypoglycemia has an effect only when amino acids or other substances found in blood, are present. In absence of the tonic effect of insulin, somatostatin is the only endogenous inhibitor of glucagon release and insulin exerts a strong inhibitory effect on the α-cell. Therefore, when an antagonist blocks the α-cell receptors, despite the inhibitory effect of injected insulin, the α-cell can release normal amounts of glucagon (Vranic, 2010). The figure is modified from that we previously reported (Vranic, 2010).

**Figure 3:**
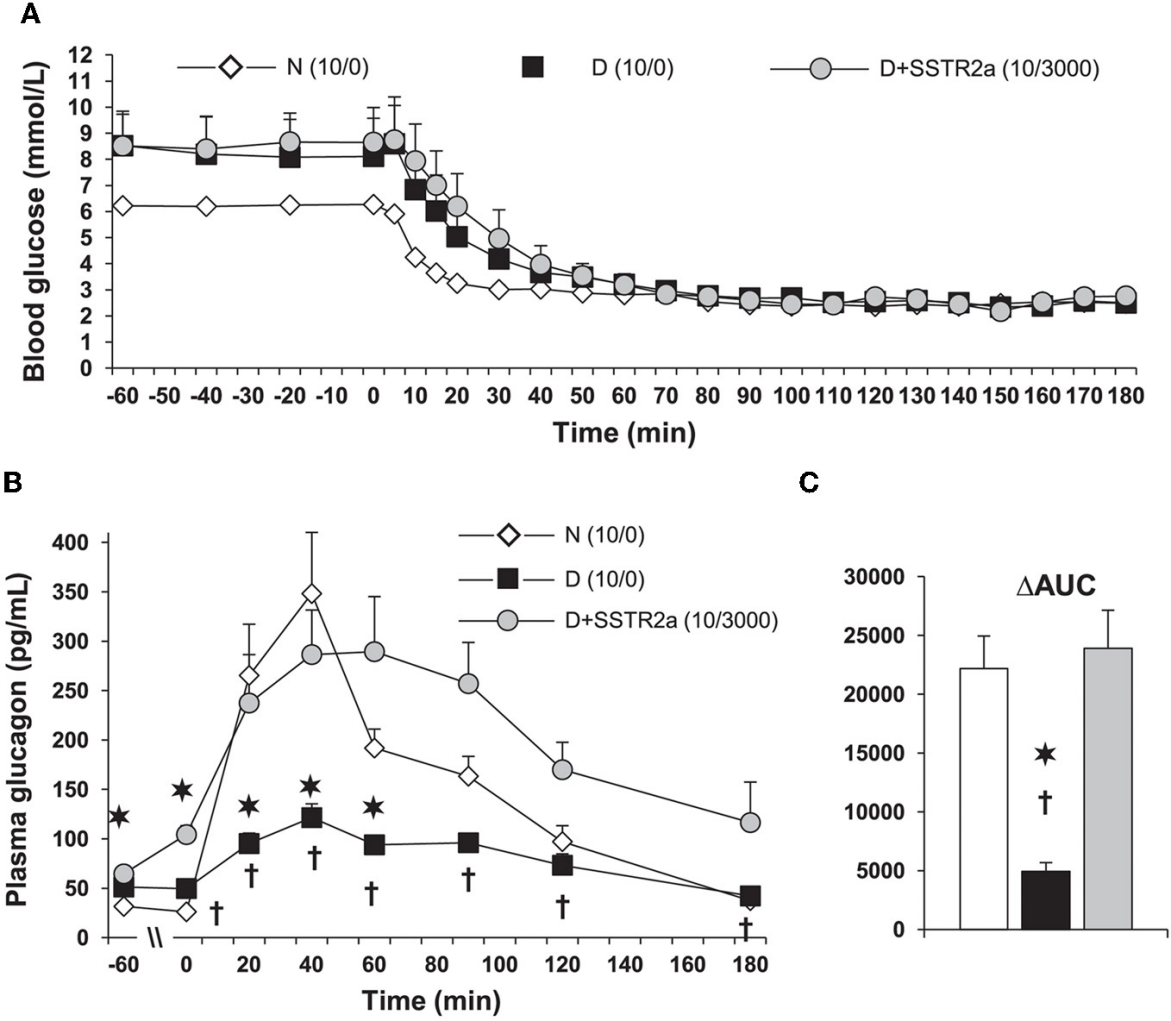
**In diabetic (D) rats, plasma glucagon increases only marginally during a glucose clamp at 2.5 mmol/L. (A)** Hypoglycemia was induced with 10 units/kg of regular insulin was injected subcutaneously. 3000 nmol/kg/h of somatostatin receptor type 2 antagonist (SSTR2a) was infused intravenously starting 30 min before insulin injection (D + SSTR2a). **(B)** The response of glucagon to hypoglycemia was the same as in normal (N) rats. **(C)** The data is also shown as area under the curve (AUC) analysis. The data is modified from that we reported in ref. Yue et al. (2011). The SSTR2a is highly specific for glucagon and only marginally for insulin, and it's structure is H-Fpa-cyclo[DCys-PAL-DTrp-Lys-Tle-Cys]-Nal-NH2 (Yue et al., 2011). ^*^P < 0.002 D vs. N; ^†^*P* < 0.05 D vs. D+SSTR2a.

Plasma somatostatin, pancreatic prosomatostatin mRNA and somatostatin protein levels are increased in diabetic humans (Orci et al., 1976), dogs (Rastogi et al., 1990), and rodents (Shi et al., 1996; Inouye et al., 2002), which may be due to insulin deficiency (Papachristou et al., 1989; Rastogi et al., 1990), although, insulin treatment did not prevent the abundance of somatostatin-containing δ-cells in human diabetic pancreata (Patel, 1999), and/or absolute or relative glucagon excess within the pancreatic islet might also lead to a compensatory increase in somatostatin (Patel, 1999). In T1D, upper-gut somatostatin, the major source of circulating somatostatin, is also increased (Papachristou et al., 1989; Patel, 1999). It is generally believed that somatostatin only plays a minor role in inhibiting the α-cell in non-diabetic animals or humans. Global somatostatin knock-out increased nutrient stimulated, but not basal glucagon secretion, compared with wild-type mice, *in-vivo* and in isolated islets, suggesting a role of locally released somatostatin on stimulated, but not basal insulin secretion (Hauge-Evans et al., 2009). Similarly, isolated islets from somatostatin receptor type-2 (SSTR2) knock-out mice showed 2-fold greater stimulated glucagon secretion than wild type mice (Strowski et al., 2000). In human isolated islets a dose-dependent reversal of SSTR2 antagonist induced suppression of glucagon secretion was achieved by using the same SSTR2 as the current study (Singh et al., 2007). Thus, using the SSTR2 antagonists may appear to also be relevant to humans. Since most β-cells have been destroyed, somatostatin becomes the main paracrine inhibitor of the α-cell in diabetes. That is why it was of particular interest that in diabetic dog islets, the ratio of somatostatin to glucagon is markedly increased. An acute insulin injection increased this ratio further. This was the first demonstration that part of the defective mechanism in hypoglycemia may reflect alterations of this ratio in diabetes (Rastogi et al., 1990). One could hypothesize that in diabetes, in absence of the tonic effect of insulin, islet α-cells are oversensitive to insulin and are exposed to increased somatostatin (Papachristou et al., 1989; Shi et al., 1996). Somatostatin is increased in the pancreas and also in blood. The major part of the concentration of somatostatin in blood is due to somatostatin release from the gut. Thus, the increase in local somatostatin and release of somatostatin delivery to the pancreas may both play a role in diabetes (Figure 2). It was previously demonstrated that in perifused islets and in infused isolated pancreas that the SSTR antagonist can greatly increase the response of α-cells to arginine. However, responses to insulin-induced hypoglycemia have not been tested. In order to test the hypothesis about the importance of somatostatin in diabetic rats, a specific antagonist (SSTR2) of the somatostatin receptor of α-cells was injected. It was demonstrated that infusion of this antagonist can fully normalize glucagon responses to insulin-induced hypoglycemia in diabetic rats [Figure 3, from ref. Yue et al. (2011)]. A patent (Vranic et al., 2009) was filed for the prevention of hypoglycemia, and hope that the results obtained in the rats can be applied to human diabetics and thereby, diminish or prevent hypoglycemic episodes. This could permit diabetic patients to adhere more strictly to an intensive insulin treatment and lessen the risk of diabetic complications.

Most importantly, infusion of the SSTR2 antagonist in absence of insulin did not affect the blood concentration of insulin, glucagon, epinephrine, or blood-sugar (Yue et al., 2011). The efficacy of the SSTR2 antagonist with two different doses of the antagonist and of insulin was tested. It was found that the most consistent results were obtained with 10 units/kg of insulin and 3000 nmol/kg/h of SSTR2 antagonist (Figure 3) (Yue et al., 2011). It is particularly interesting that in normal rats the antagonist did not improve or even decrease the response of glucagon to insulin-induced hypoglycemia. One could speculate that in normal rats, the high doses of antagonist even have some agonist properties, and confirmed that in normal rats, somatostatin is not a major inhibitor of hypoglycemia-induced glucagon release. The response of corticosterone was also normalized. Corticosterone in contrast to glucagon is important for hypoglycemias of longer duration, since the effects of cortisol are mainly exerted through genetic mechanisms. This could also be of importance for glucagon release because cortisol has some effect on the α-cells' control. Interestingly, delivery of the SSTR2 antagonist did not further increase pancreatic glucagon and somatostatin, or plasma somatostatin.

One of the key questions was whether the SSTR2 antagonist can actually prevent hypoglycemia. On the first day insulin alone, and on the second day, either insulin alone or an infusion of antagonist was started in the same rat, before the insulin-induced hypoglycemia (Vranic et al., 2009). The reason for such designs is that even one episode of hypoglycemia sensitizes the endocrine and metabolic system so that you would expect that on the second day the rats would need a different amount of insulin. In order to avoid this problem, diabetic rats were injected for 3 days with insulin, in order to avoid further effect of antecedent hypoglycemia. After the injection of insulin, rats became hypoglycemic, but with the SSTR2 antagonist, hypoglycemia was avoided. Without the antagonist, glucagon response was abolished, but with the antagonist, glucagon response was restored (Yue et al., 2010). These STZ-induced diabetic rats were not treated with insulin since they still have some residual insulin in the blood and in the pancreas. In contrast, BB rats are totally insulin-deprived, thus requiring insulin treatment, and therefore this model is more similar to human T1D; both caused by immune destruction of the β-cells. The *in-vivo* to *in-vitro* responses to hypoglycemia and arginine in controls and in diabetic BB rats were compared (Qin et al., 2012). In the *in-vivo* experiments, the glucose was clamped at 2.9 mmol/L. In contrast to the controls, the glucagon response was greatly diminished, but it was normalized during the infusion of the SSTR2 antagonist. With glucagon response normalized, the BB rats did not need glucose infusion to maintain the clamp, while without the antagonist they needed a large amount of glucose infused because of the glucagon deficiency. Interestingly, we used for the first time pancreatic slices to assess the effect of hypoglycemia and arginine. Surprisingly, hypoglycemia *per se* did not increase glucagon release. However, glucagon release was enhanced when arginine was infused (Qin et al., 2012). The difference between *in-situ* and *in-vitro* experiments is that pancreatic slices are not controlled by the nervous system and are not exposed to hormones or metabolites (such as, arginine) that stimulate glucagon release.

It was questioned whether somatostatin plays a role during hypoglycemia because somatostatin-secreting δ-cells are downstream of glucagon-secreting α-cells in the islet microcirculation of non-diabetic rats (Samols et al., 1988). However, δ-cells in diabetic rats are also distributed in central portions of islet cells because the architecture of islet cell type is altered (Adeghate, 1999), suggesting that paracrine actions of islet hormones are altered in diabetes such that somatostatin release upstream of α-cells may affect glucagon secretion. The arrangement of human endocrine islet cells is likewise more disperse throughout the islet, which provides evidence for the proximity of δ-cells and α-cells (Cabrera et al., 2006; Braun et al., 2009; Kim et al., 2009). Furthermore, paracrine signaling may also occur via diffusion within the islet interstitium, independent of blood flow.

The remaining question to be answered is to explore factors in blood that are necessary to sensitize the responses of α-cell to hypoglycemia and the mechanism of the potential sensitization of α-cells to insulin in diabetes. These results indicate that SSTR2 blockade (Rossowski et al., 1998; Hocart et al., 1999) may be a novel treatment strategy of improving counterregulatory responses, including glucagon, to insulin-induced hypoglycemia. This strategy could lead to prevention of hypoglycemia in insulin-treated diabetics.

## Future directions

Considerable work investigating glucagon secretion and α-cell signaling in healthy islets have been done as discussed above. However, there has been relatively little progress in assessing the perturbation of α-cellular physiology and paracrine dysregulation during diabetes, which will require more innovative approaches. One approach is the pancreatic slice preparation (Huang et al., 2011a,b) whereby the α-cell and its precise secretory physiology within intact pancreatic tissue could be examined by patch-clamp technique, and perhaps later could also be further assessed by imaging. The slice preparation has very recently enabled us to begin to assess α-cell dysfunction in T1D wherein the very small islet mass and inflammation would have rendered it impossible to reliably isolate and examine the α-cell (Huang et al., 2012). In that report, α-cells in STZ-induced diabetic mice exhibited enhanced Na^+^ currents and reduced Kv currents, contributing to the increased action potential amplitude and firing frequency. This, along with the larger glucagon granules (found on E.M.) carrying larger amount of glucagon cargo, would trigger more glucagon release, thus explaining the basis of hyperglucagonemia in T1D (Huang et al., 2012). Future studies employing the pancreas slice preparation will enable the elucidation of paracrine regulation within normal and diabetic islets. Another approach is genetic manipulation of candidate proteins within α-cells by α-cell-specific knockout mouse models (Gustavsson et al., 2009; Kawamori et al., 2009) and genetic β-cell ablation models mimicking T1D (Thorel et al., 2010). Ideally, these clever approaches could be combined.

From a clinical point of view, the mechanism whereby in T2D there is excessive response to glucagon during meals, and whether pharmacological intervention can prevent this problem. A key question is also whether it is possible to prevent hypoglycemia in insulin-treated diabetics. So far, the evidence was obtained only in STZ-treated and BB rats.

### Conflict of interest statement

Patrick E. MacDonald receives research funding for his work on α-cells from Merck. Herbert Y. Gaisano and Mladen Vranic have no financial or commercial relationships.

## Abbreviations

Type 1 diabetes: T1D
Type 2 diabetes: T2D
glucagon-like peptide-1: GLP-1
streptozotocin: STZ
BioBreeding: BB
glucagon receptor-null mice: *Gcgr*^−/−^
insulin-like growth factor: IGF
voltage-dependent Ca^2+^ channel: VDCC
ATP-dependent K^+^ channel: K_ATP_ channel
voltage-dependent K^+^ channel: Kv channel
AMP-activated protein kinase: AMPK
Per-arnt-sim (PAS) domain-containing protein kinase: PASK
mouse insulin promoter- green fluorescent protein: MIP-GFP
γ-aminobutyric acid: GABA
somatostatin receptor type-2: SSTR2.
